# Supplementary material for: Discovery of a Unique Set of Dog-Seroreactive Coccidioides Proteins Using Nucleic Acid Programmable Protein Array
Source: J Fungi (Basel). 2024 Apr 24;10(5):307. doi: 10.3390/jof10050307 (PMC11121964; doi:10.3390/jof10050307)
Supplement: Supplementary file 1 [file jof-10-00307-s001.zip › jof-2935619-supplementary.pdf]

Supplemental Materials

Naturally Infected dogs (N=17)				
	Seropositivity		Odds Ratio	p-value
Protein	Positive	Negative	Positive	Positive
CF	80%	0%	400.0	1.44E-03
CIMG_04988	60%	0%	150.0	8.52E-03
CIMG_05828	60%	0%	150.0	8.52E-03
CIMG_02401	30%	0%	42.9	3.89E-02
CIMG_04729	30%	0%	42.9	3.89E-02
CIMG_11455	30%	0%	42.9	3.89E-02
CIMG_10136	20%	0%	25.0	5.34E-02
CPSG_06799	20%	0%	25.0	5.34E-02
CIMG_00220	10%	0%	11.1	6.84E-02
CIMG_06562	10%	0%	11.1	6.84E-02
CIMG_06738	10%	0%	11.1	6.84E-02
CIMG_07827	10%	0%	11.1	6.84E-02
CIMG_10149	10%	0%	11.1	6.84E-02
CIHG_08476	10%	0%	11.1	6.84E-02
CIMG_12861	40%	0%	66.7	2.61E-02
CIMG_07322	30%	0%	42.9	3.89E-02
CIMG_05911	20%	0%	25.0	5.34E-02
CIMG_06723	20%	0%	25.0	5.34E-02
CIMG_00627	10%	0%	11.1	6.84E-02
CIMG_00965	10%	0%	11.1	6.84E-02
CIMG_01310	10%	0%	11.1	6.84E-02
CIMG_01552	10%	0%	11.1	6.84E-02
CIMG_01749	10%	0%	11.1	6.84E-02
CIMG_02699	10%	0%	11.1	6.84E-02
CIMG_03708	10%	0%	11.1	6.84E-02
CIMG_05579	10%	0%	11.1	6.84E-02
CIMG_06320	10%	0%	11.1	6.84E-02
CIMG_06998	10%	0%	11.1	6.84E-02
CIMG_07263	10%	0%	11.1	6.84E-02
CIMG_09143	10%	0%	11.1	6.84E-02
CIMG_09691	10%	0%	11.1	6.84E-02
CIMG_12767	10%	0%	11.1	6.84E-02
CIMG_13032	10%	0%	11.1	6.84E-02

Table S1. Naturally Infected Dogs Table. This table shows the seropositivity, Odds ratio, as well as the p-value for the naturally infected dogs (N=17). Seropositivity goes from dark blue (0%) to dark red (100%), odds-ratios go from light blue (0) to dark red (>10), with p-values highlighted in green if they are significant (<0.05).

Laboratory Infected Dogs (N=18)													
	Seropositivity					Odds Ratio				p-values			
Protein	C0	C2	C4	C6	C8	C2	C4	C6	C8	C2	C4	C6	C8
CF	2%	0%	81%	###	###	0	191	###	###	2.6E-01	3.7E-13	3.2E-15	1.6E-12
CIMG_04988	2%	0%	50%	71%	82%	0	44	110	198	2.6E-01	1.3E-07	3.7E-10	6.5E-10
CIMG_05828	2%	0%	6%	29%	45%	0	3	18	37	2.6E-01	1.2E-01	6.7E-04	3.2E-05
CIMG_02401	0%	0%	6%	21%	18%	1	7	27	22	2.8E-01	3.1E-02	6.3E-04	4.9E-03
CIMG_04729	2%	7%	31%	29%	27%	3	20	18	17	1.7E-01	8.6E-05	6.7E-04	3.8E-03
CIMG_11455	2%	0%	6%	7%	18%	0	3	3	10	2.6E-01	1.2E-01	2.2E-01	3.5E-02
CIMG_10136	7%	0%	13%	14%	27%	0	2	2	5	1.5E-01	1.3E-01	2.1E-01	4.8E-02
CPSG_06799	0%	47%	75%	79%	64%	88	300	367	175	8.4E-08	4.2E-13	1.7E-12	1.5E-08
CIMG_00220	2%	7%	13%	0%	18%	3	6	0	10	1.7E-01	2.6E-02	3.8E-01	3.5E-02
CIMG_06562	0%	33%	19%	14%	36%	50	23	17	57	8.0E-06	5.4E-04	5.6E-03	3.7E-05
CIMG_06738	9%	27%	13%	14%	36%	4	1	2	6	2.9E-02	1.7E-01	3.1E-01	2.0E-02
CIMG_07827	7%	27%	19%	21%	36%	5	3	4	8	1.2E-02	4.4E-02	6.0E-02	7.6E-03
CIMG_10149	0%	13%	13%	7%	18%	15	14	8	22	5.0E-03	4.1E-03	5.0E-02	4.9E-03
CIHG_08476	0%	20%	19%	0%	27%	25	23	1	38	6.1E-04	5.4E-04	4.0E-01	4.4E-04
CIMG_12861	11%	0%	25%	14%	0%	0	3	1	0	8.5E-02	4.8E-02	3.9E-01	2.9E-01
CIMG_07322	2%	0%	13%	14%	0%	0	6	7	0	2.6E-01	2.6E-02	3.8E-02	7.8E-01
CIMG_05911	0%	0%	6%	0%	0%	1	7	1	1	2.8E-01	3.1E-02	4.0E-01	7.3E-01
CIMG_06723	7%	13%	25%	7%	0%	2	5	1	0	1.7E-01	1.1E-02	4.3E-01	4.5E-01
CIMG_00627	0%	0%	0%	0%	9%	1	1	1	10	2.8E-01	1.9E-01	4.0E-01	5.7E-02
CIMG_00965	4%	0%	0%	7%	18%	0	0	2	5	2.0E-01	1.3E-01	3.7E-01	1.1E-01
CIMG_01310	0%	7%	0%	7%	0%	7	1	8	1	4.1E-02	1.9E-01	5.0E-02	7.3E-01
CIMG_01552	0%	0%	0%	0%	9%	1	1	1	10	2.8E-01	1.9E-01	4.0E-01	5.7E-02
CIMG_01749	4%	0%	0%	7%	18%	0	0	2	5	2.0E-01	1.3E-01	3.7E-01	1.1E-01
CIMG_02699	2%	7%	0%	0%	9%	3	0	0	4	1.7E-01	1.7E-01	3.8E-01	2.7E-01
CIMG_03708	0%	0%	6%	0%	0%	1	7	1	1	2.8E-01	3.1E-02	4.0E-01	7.3E-01
CIMG_05579	0%	7%	6%	7%	9%	7	7	8	10	4.1E-02	3.1E-02	5.0E-02	5.7E-02
CIMG_06320	0%	7%	0%	0%	0%	7	1	1	1	4.1E-02	1.9E-01	4.0E-01	7.3E-01
CIMG_06998	0%	0%	0%	0%	9%	1	1	1	10	2.8E-01	1.9E-01	4.0E-01	5.7E-02
CIMG_07263	0%	0%	6%	0%	0%	1	7	1	1	2.8E-01	3.1E-02	4.0E-01	7.3E-01
CIMG_09143	4%	7%	19%	7%	18%	2	5	2	5	2.7E-01	1.8E-02	3.7E-01	1.1E-01
CIMG_09691	7%	13%	0%	0%	9%	2	0	0	1	1.7E-01	9.6E-02	2.2E-01	7.8E-01
CIMG_12767	2%	13%	0%	0%	9%	7	0	0	4	3.3E-02	1.7E-01	3.8E-01	2.7E-01
CIMG_13032	0%	7%	6%	0%	0%	7	7	1	1	4.1E-02	3.1E-02	4.0E-01	7.3E-01

Table S2. Laboratory Infected Dogs Table. This table shows the seropositivity, Odds ratio, as well as the p-value for the laboratory infected dogs (N=18). Values are shown for each timepoint. Seropositivity goes from dark blue (0%) to dark red (100%), odds-ratios go from light blue (0) to dark red (>10), with p-values highlighted in green if they are significant (<0.05).

Vaccinated + Challenged Dogs (N=6)													
Vaccine	Seropositivity					Odds Ratio				p-values			
Protein	V0	V2	V4	V6	C0	V2	V4	V6	C0	V2	V4	V6	C0
CF	0%	0%	50%	67%	33%	1	10	20	5	7.5E-01	5.6E-02	1.8E-02	1.5E-01
CIMG_01749	0%	0%	17%	33%	50%	1	2	5	10	7.5E-01	3.7E-01	1.5E-01	5.6E-02
CIMG_04729	0%	0%	17%	50%	0%	1	2	10	1	7.5E-01	3.7E-01	5.6E-02	7.5E-01
CIMG_04988	0%	17%	50%	67%	67%	2	10	20	20	3.7E-01	5.6E-02	1.8E-02	1.8E-02
CIMG_05828	0%	0%	17%	17%	0%	1	2	2	1	7.5E-01	3.7E-01	3.7E-01	7.5E-01
CIMG_10136	0%	17%	17%	33%	33%	2	2	5	5	3.7E-01	3.7E-01	1.5E-01	1.5E-01
CIMG_11455	0%	17%	33%	33%	33%	2	5	5	5	3.7E-01	1.5E-01	1.5E-01	1.5E-01
CPSG_05795	0%	50%	33%	83%	67%	10	5	50	20	5.6E-02	1.5E-01	4.2E-03	1.8E-02
Challenge		Seropositivity				Odds Ratio				p-values			
Protein		C2	C4	C6	C8	C2	C4	C6	C8	C2	C4	C6	C8
CF		33%	50%	50%	67%	5	10	10	20	1.5E-01	5.6E-02	5.6E-02	1.5E-01
CIMG_01749		0%	17%	33%	17%	1	2	5	2	7.5E-01	3.7E-01	1.5E-01	3.7E-01
CIMG_04729		0%	17%	17%	17%	1	2	2	2	7.5E-01	3.7E-01	3.7E-01	3.7E-01
CIMG_04988		33%	50%	67%	50%	5	10	20	10	1.5E-01	5.6E-02	1.8E-02	5.6E-02
CIMG_05828		0%	17%	17%	17%	1	2	2	2	7.5E-01	3.7E-01	3.7E-01	3.7E-01
CIMG_10136		17%	17%	33%	17%	2	2	5	2	3.7E-01	3.7E-01	1.5E-01	3.7E-01
CIMG_11455		33%	33%	17%	17%	5	5	2	2	1.5E-01	1.5E-01	3.7E-01	3.7E-01
CPSG_05795		83%	67%	83%	67%	50	20	50	20	4.2E-03	1.8E-02	4.2E-03	1.8E-02

Table S3. Vaccinated + Challenged Dogs Table. This table shows the seropositivity, Odds ratio, as well as the p-value for the vaccinated + challenged dogs (N=6). Values are shown for each timepoint. Seropositivity goes from dark blue (0%) to dark red (100%), odds-ratios go from light blue (0) to dark red (>10), with p-values highlighted in green if they are significant (<0.05).
